# Simultaneous measurements of velocity, oxygen concentration, and deformed interface position in an air–water channel using PIV and LIF

**DOI:** 10.1007/s00348-025-04017-w

**Published:** 2025-04-18

**Authors:** Adharsh Shankaran, R. Jason Hearst

**Affiliations:** Department of Energy and Process Engineering, Norwegian University of Science and Technology, Gløshaugen, 7032 Trondheim, Norway

## Abstract

Oxygen transfer across a deforming air–water interface is studied using a synergy of particle image velocimetry and laser-induced fluorescence (LIF). Such approaches have previously been limited to flat interfaces. We develop simultaneous measurements of velocity fields, dissolved oxygen (DO) concentration fields, and interface positions for spatial and temporal tracking. The imaging process begins after the DO in the water has been chemically depleted and continues until the water is saturated with DO. The oxygen LIF intensity field is calibrated using measurements from an optical oxygen probe to ensure accurate conversion into physical unit (mg/L). A canonical air turbulent channel flow, with a centerline velocity of 6.6 m/s (Reynolds number based on channel height of 21,700), develops for more than 100 heights before the bottom boundary condition is changed from a solid wall to a water surface. This induces transient and wavy structures on the air–water interface and generates velocity fluctuations and vorticity on the water side, which drives DO transport. The spatial evolution of DO concentration reveals steep gradients near the interface that diminish with depth, while the temporal evolution shows a reduction in concentration differences between the bulk and interface from about 35% to less than 5% as the water saturates. Concentration fluctuations are lower near the interface compared to the bulk and diminish in time as the system approaches saturation. Turbulent scalar transport analysis shows high vertical flux near the interface, and this too changes as the bulk DO concentration evolves, emphasizing that the observed phenomena are transient and should be treated as such.

## Introduction

### Motivation

Gas exchange at the air–water interface is a crucial phenomenon with significant implications for various industrial and environmental applications. For instance, dissolved oxygen (DO) levels in water impact the efficiency of boilers due to corrosion (Kobe and Gooding 1935), while the re-aeration rate of water affects its quality and ecological health. Furthermore, oxygen ($\hbox {O}_2$) and carbon dioxide ($\hbox {CO}_2$) fluxes between the atmosphere and the ocean play a vital role in the global carbon cycle and climate change dynamics (Gruber et al. 2019; Li et al. 2020).

Accurately measuring concentration gradients and fluxes of dissolved gases remains a significant challenge in studying gas exchange at the air–water interface. The gas transfer rate depends on the interplay between molecular diffusion and turbulence near the interface (Jirka et al. 2010). This effect is particularly pronounced for low-solubility gases like oxygen, where exchange is primarily controlled by molecular diffusion across the diffusive boundary layer.

### Literature review

The surface renewal model (SRM) provides a framework for describing the gas transfer process by relating the gas transfer velocity, *k*, to the large eddy turnover time, given by $k \sim (D/\tau )^{1/2}$, where *D* is the molecular diffusivity and $\tau$ represents the large eddy turnover time. The latter can be approximated as $\tau \sim u_1'/L$, where $u_1'$ is the standard deviation of turbulent fluctuations and *L* is the integral length scale (Higbie 1935; Danckwerts 1951; Fortescue and Pearson 1967). Surfactants add further complexity by suppressing gas transfer and altering interfacial dynamics (Mölder et al. 2002; Pereira et al. 2018). These effects must be carefully considered when modeling and measuring air–water gas exchange processes.

Under low to moderate wind conditions, when wave breaking is absent, small-scale near-surface turbulence is an important parameter, and *k* can alternatively be parameterized by the small eddy model (SEM). This model takes the form $k \sim (D(\epsilon \nu )^{1/2})^{1/2}$ (Banerjee et al. 1968; Lamont and Scott 1970), where $\epsilon$ is the mean dissipation rate of turbulent kinetic energy and $\nu$ is the kinematic viscosity.

Another model, known as the surface divergence model (SDM), developed by McCready et al. (1986), considers surface velocity divergence as the driving parameter for *k*, leading to the expression $k \sim (D \beta ')^{1/2}$, where $\beta '$ is the root mean square of surface divergence.

These various models highlight different dominant mechanisms, sometimes leading to contradictions. To reconcile these discrepancies, Theofanous et al. (1976) proposed that small eddies dominate gas transfer at high Reynolds numbers, while large eddies dominate at low Reynolds numbers. Later, Wang et al. (2015) conducted a comparative analysis of SEM and SDM to assess their ability to characterize *k*. Their findings showed correlations between *k* and both dissipation rate and surface divergence, suggesting that a single model might not be sufficient. Similarly, Turney and Banerjee (2013) developed a combined SRM-SDM model, which aligned well with measurements from open-channel and wind-driven flows.

A key reason for inconsistencies in existing models is the difficulty in obtaining accurate experimental validation. Measuring dissolved gas concentrations and velocity fields near the air–water interface requires reliable techniques. One common approach is using point probe measurements for DO alongside particle image velocimetry (PIV), as demonstrated by O’Connor and Hondzo (2008) and Sanjou (2020). However, this method captures spatial variations only at discrete points. Another approach, employed by Turney and Banerjee (2013), combines fluorescence-decay dissolved oxygen sensors with interfacial PIV. Although effective, the 5-second response time of the DO sensor limits its ability to capture instantaneous variations.

Alternative techniques such as acid–base reactions to visualize dissolved $\hbox {CO}_2$ (Münsterer and Jähne 1998; Variano and Cowen 2013; Lacassagne et al. 2017) have also been explored. However, these methods are unsuitable near the interface in advecting flows due to the relatively long reaction times of the process. To address these limitations, quenching-based methods have been developed for instantaneous DO concentration measurements with spatial resolution. Notable works (Duke and Hanratty 1995; Münsterer et al. 1995; Herlina and Jirka 2004; Jirka et al. 2010) have employed 1-pyrene butyric acid (PBA) for this purpose. Originally introduced for biological research (Vaughn and Weber 1970), PBA exploits oxygen as a collisional quencher, reducing fluorescence lifetime and enabling rapid DO measurements. Herlina and Jirka (2008) further advanced this technique by integrating PBA-based DO measurements with PIV in an oscillating grid turbulence tank with no mean flow.

### Objectives

The primary objective of this paper is to achieve simultaneous measurements of velocity and DO concentration fields under realistic conditions, including wind-sheared flows, deformable water surfaces, and advection. Such measurements have not been previously achieved.

Section 2 provides a comprehensive description of the experimental setup and measurement techniques. Section 3 details the calibration approach. The results, presented in Sect. 4, focus on an advecting flow case with a deformable interface, highlighting key flow features and DO evolution from near-zero concentration to saturation. Finally, Sects. 5 and 6 discuss challenges for future researchers and present the general conclusions of the study.

## Methodology

### Experimental setup

**Fig. 1:**
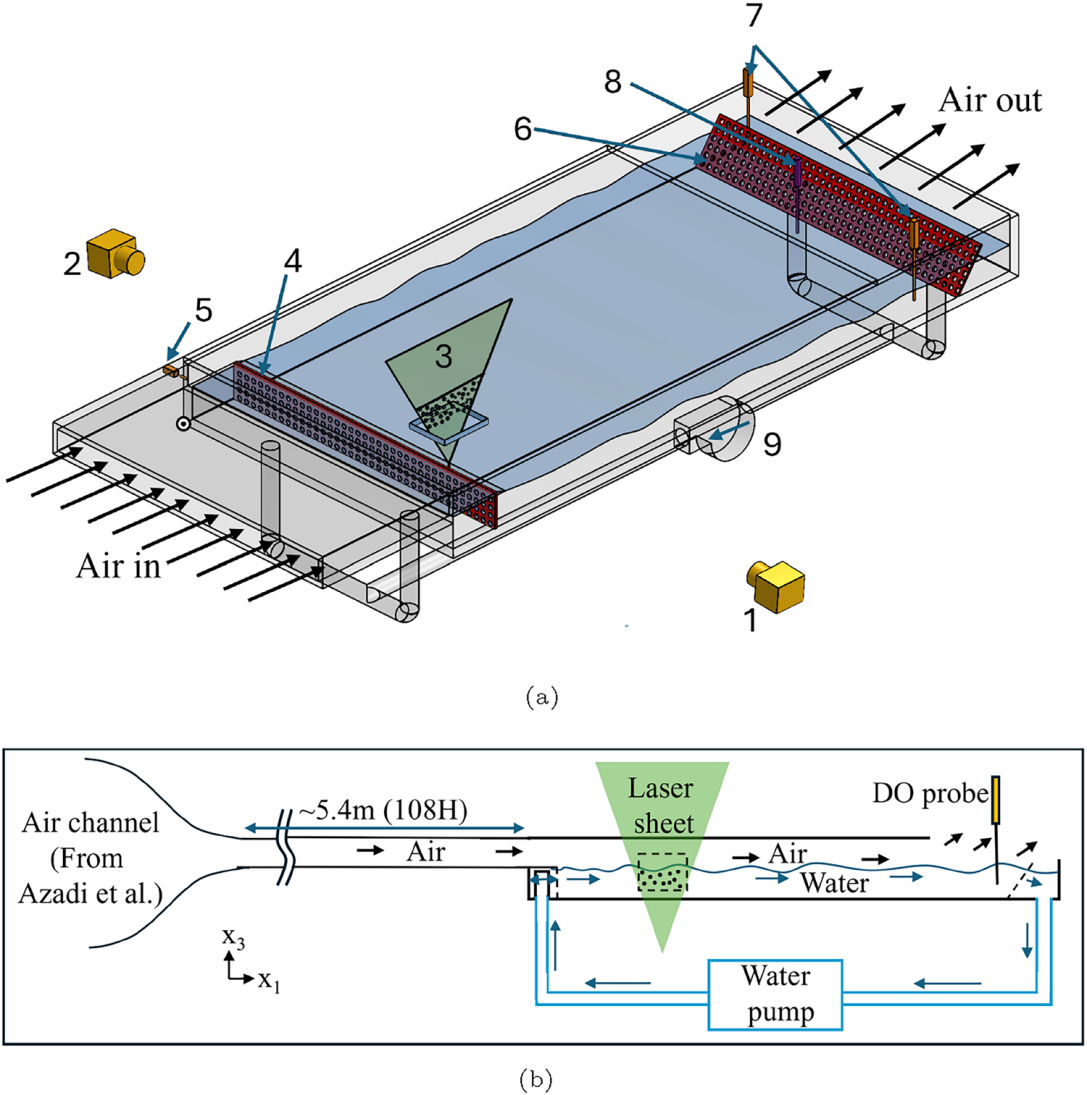
**a** Schematic of the air–water channel. 1. PIV camera; 2. camera for oxygen LIF; 3. laser sheet with both 532 nm and 266 nm wavelengths; 4. inlet flow conditioner; 5. thermocouple (air); 6. extruded mesh; 7. thermocouple (water); 8. oxygen probe; 9. water pump.** b** Side view schematic of air–water channel

The experiments are conducted in an air–water channel flow facility with dimensions of 1300 mm (length), 600 mm (width), and 106 mm (height), constructed from acrylic. A schematic of the setup is shown in Fig. 1. This facility is positioned downstream of a turbulent air channel, which measures 5425 mm in length, 600 mm in width, and 50 mm in height, as described by Asadi et al. (2022). In this configuration, the turbulent airflow evolves over approximately 108 channel heights before interacting with the water surface. By this point, the first- and second-order statistics of the flow have achieved a self-similar state, ensuring well-developed turbulence for the experiments.

The air–water channel is filled with water to a height of 50 mm (*H*), leaving the remaining space above for airflow. A pump is used to generate a circulating water flow within the channel. Meshes are installed at the channel inlet to condition the water flow. Additionally, angled extruded meshes are placed at the channel’s outlet to dampen waves and minimize wave reflections, ensuring smooth flow conditions for the experiments.

A quartz glass window, positioned at the bottom of the channel and 275 mm (5.5*H*) downstream of where the water and air start to interact, facilitates the transmission of the co-located laser sheets at wavelengths of 266 nm and 532 nm. A 125 mm horizontal plate is positioned at the inlet, elevated 50 mm above the water channel base, and touching the water surface. It minimizes ripples caused by the water flow at the inlet, ensuring that all surface deformations result only from the interaction between the airflow and the water, rather than the water pumping mechanism. The plate’s end is angled to prevent boundary layer separation of the air at the inlet.

### Measurements in water

A Litron Nano PIV laser, capable of generating co-located beams at 532 nm (216 mJ) and 266 nm (30 mJ) simultaneously, is used for PIV and oxygen laser-induced fluorescence (LIF) measurements in water. The laser beam passes through a LaVision energy monitor and a mirror before entering the measurement area via LaVision sheet optics. The water is seeded with 20 $\mu$m polystyrene Dynoseed particles for PIV.

Dissolved oxygen concentration is measured using LIF with 1-pyrenebutyric acid (PBA, CAS: 3443-45-6) as a fluorescent tracer. The PBA is dissolved in 0.1 N sodium hydroxide solution and diluted with water to achieve a $2.6 \times 10^{-6}$ M solution. When excited by wavelengths between 250 and 350 nm, PBA fluoresces between 370 and 410 nm. According to the Stern–Vollmer equation, the fluorescence intensity of PBA which is proportional to fluorescence lifetime is related to oxygen concentration as

1 $$\begin{aligned} \frac{I_o}{I}= \frac{\tau _o}{\tau }=1+K_{sv}Q, \end{aligned}$$

where $I_o$ and $\tau _o$ represent the intensity and fluorescence lifetime in the absence of dissolved oxygen, respectively, $K_{sv}$ is the Stern–Vollmer constant, *Q* is the dissolved oxygen concentration, and *I* and $\tau$ the intensity and fluorescence lifetime at any instant.

Before introducing PBA, sodium sulfite ($\hbox {Na}_2$ $\hbox {SO}_3$) is used as an oxygen scavenger to deoxygenate the water, as detailed by Sanjou (2020). The laser sheet is directed into the water flow through the bottom quartz window, effectively illuminating the flow field and the air–water interface.

A two-camera system, consisting of a LaVision sCMOS camera (2560 pixels $\times$ 2160 pixels resolution, 16-bit depth) for oxygen LIF and a LaVision Imager LX 16 M camera (4920 pixels $\times$ 3280 pixels resolution, 12-bit depth) for PIV, is utilized, as shown in Fig. 1. Images are taken in double frame mode with an inter-frame time of 3 ms. The PIV camera is positioned at an upward angle of 3 degrees to record PIV particles, while the oxygen LIF camera is placed on the opposite side of the air–water channel, also at an upward angle of approximately 3 degrees. Both cameras are equipped with Sigma 105 mm lenses.

The PIV camera has a field of view of approximately 60 mm (width) $\times$ 35 mm (height), while the oxygen LIF camera has a field of view of about 37 mm (width) $\times$ 35 mm (height). Since the cameras are positioned at a slight upward angle to prioritize the air–water interface, the region above the air–water interface in the image is covered with reflections. A 532 nm centered narrow bandpass filter is attached to the PIV camera, while a 390 nm centered bandpass filter is attached to the oxygen LIF camera, ensuring that each camera captures only the desired wavelength for its respective technique.

Three thermocouples (one at the air inlet and two in water) are utilized to monitor the temperature throughout the experiment. A PreSens optical oxygen point probe is placed at approximately 22*H* from the start and around 6*H* from the side of the water channel and approximately 0.5*H* depth of water to monitor the bulk oxygen concentration. The approximate position of the probes is shown in the schematic Fig. 1.

### Camera calibrations

The cameras were calibrated using a LaVision calibration plate (3D plate of type: 058-5). Once the water channel is filled with deionized (DI) water to the required height, calibration images were acquired for PIV and the oxygen LIF camera. DaVis (version 10.2) was used to perform a polynomial calibration, which was applied to all images. All cameras recorded at 0.5 Hz throughout the experiment to capture the mechanics and statistics as oxygen dissolves into water over the entire period from zero DO concentration to full saturation. The experiment lasted approximately 147 min and captured 4410 image pairs.

### Air–water interface identification and PIV

**Fig. 2:**
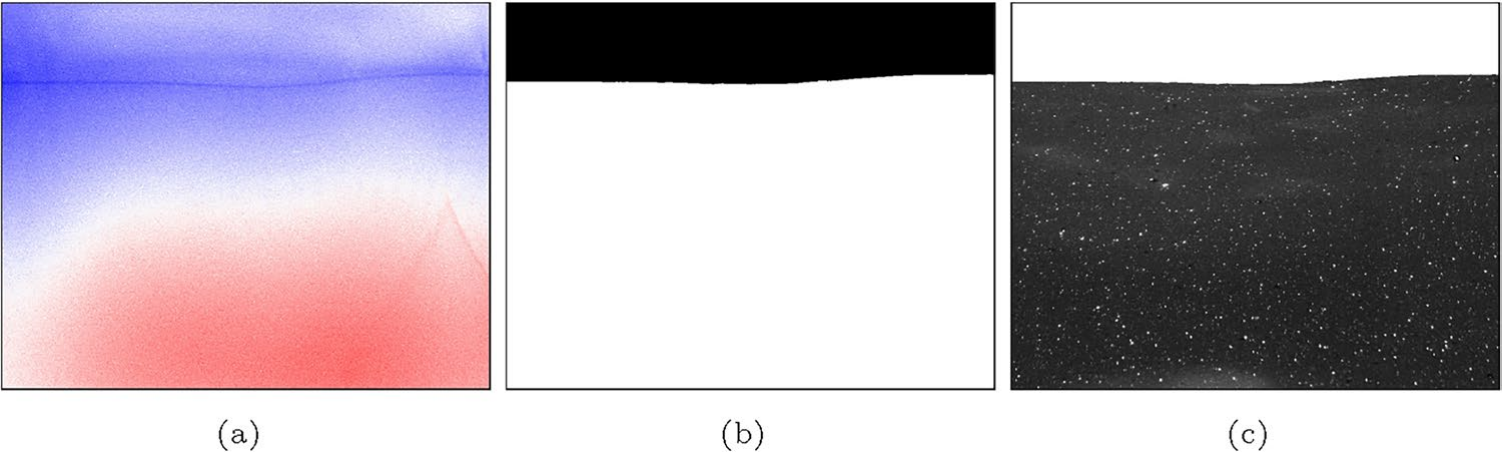
**a** Raw oxygen LIF image,** b** mask extracted from the oxygen LIF image,** c** mask applied to PIV image

The perspective-corrected images from the oxygen LIF camera were used to identify the air–water interface, which appears as a dark line in the images because of the high DO concentration at the interface. A search algorithm is employed on each vertical array of the image to locate this dark line, with the minimum intensity within this line being selected as the interface. A binary mask is then generated, which is applied to both the oxygen LIF and PIV images for accurate surface identification; see Fig. 2. To improve the signal-to-noise ratio, the particle images for PIV are further filtered by subtracting a sliding filter with a length of 10 pixels. PIV analysis is performed on these filtered images, starting with an initial pass of $128\times 128$ pixel square window with an overlap of 50% followed by four final passes with a $64\times 64$ pixel adaptive window with an overlap of 50%. This results in one vector every 0.29 mm. The adaptive window is particularly useful due to the presence of a deformed air–water interface.

### Hot-wire measurements in air

Hot-wire measurements were performed to measure the statistical properties of the incoming turbulent flow. A single-wire probe from Dantec Dynamics (model number 55P11) was employed. The hot wire was placed immediately upstream of the water channel, i.e., at a distance of 5425 mm (approximately 108 air channel heights) from the start of the air channel, in the center of the section. The measured velocity is used to calculate the inlet Reynolds number, and the Taylor and Kolmogorov scales of incoming flow to the air–water section; these quantities are discussed further in Sect. 4.1. An overheat ratio of 1.8 was employed with a sampling frequency of 75 kHz using a DISA M-series constant temperature anemometer. The output signal was filtered by a Schrödinger 25 kHz analog filter to remove high-frequency noise and prevent aliasing. The signal was acquired over 600 s.

## Oxygen LIF calibration

Calibration of the oxygen LIF images was performed using a PreSens optical oxygen probe, which is positioned approximately 25 mm (0.5*H*) deep in the water, at the mid-height of the channel and around 15*H* from the PIV/LIF measurement area. Operating at a frequency of 1 Hz, the probe provides measurements that represent the bulk oxygen concentration. The probe is calibrated by immersing it in water with known zero and saturation oxygen concentrations at room temperature. Zero concentration is achieved by adding 1 g of sodium sulfite to 100 mL of water, while saturation is attained by bubbling the water for 10 min and gently stirring it with a glass rod to prevent over-saturation.

**Fig. 3:**
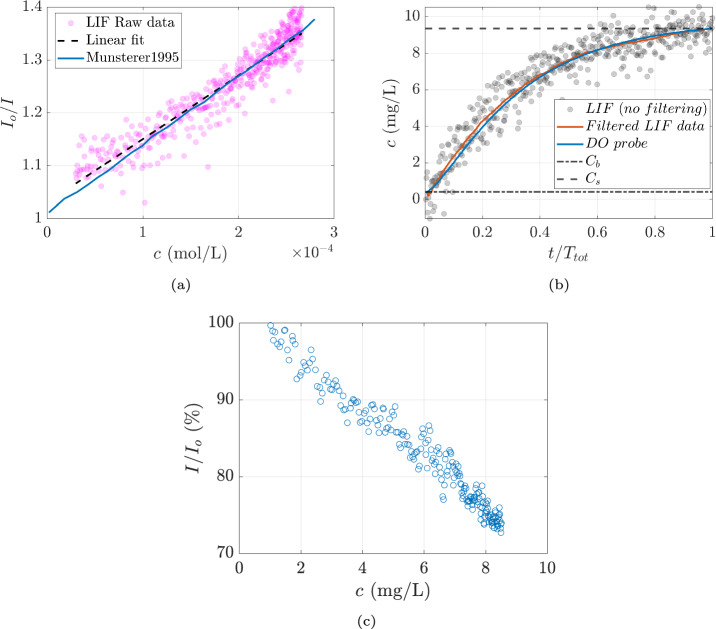
**a** Ratio of maximum intensity to instantaneous intensity is plotted against probe measurements of DO concentration. The slope of the line corresponds to $K_{sv}$ of the Stern–Vollmer equation,** b** DO concentration plotted against normalized time with $C_s$ and $C_b$ used in Eq. 3 from a test data set.** c** Relative intensity vs probe measurements indicate about 28% intensity reduction over the range of DO concentration studied. In all the plots, every 10th point is plotted for visual clarity

To verify the method, the ratio of maximum intensity ($I_o$), indicative of the lowest oxygen concentration, to the instantaneous pixel intensity (*I*) is plotted against the bulk measurements, as shown in Fig. 3a. A linear variation of $I_o/I$ is expected, as described by the Stern–Vollmer equation (Eq. 1) (Vaughn and Weber 1970). The linear fit closely matches the data from Münsterer et al. (1995). However, it should be noted that the slope value reported by Münsterer et al. (1995) is $683 \pm 70$ L/mol at 298 K, which is approximately half of the actual value observed here, where the upper and lower bounds of the slope are 1160 L/mol and 1120 L/mol, respectively, at water temperature of 285 K and air temperature of 290 K.

The spread of raw intensity data results from a combination of LIF data uncertainty and the actual physical process. Separating the contributions of different uncertainty sources is challenging; however, we apply the following methods to estimate overall uncertainty. The mean standard deviation from all the pixels, calculated from a sample set of approximately 80 images ($2.2\times 10^6$ points), was used to assess intensity variation at the same oxygen concentration, suggesting a resolution of 2.8%. This is slightly better than (Herlina and Jirka 2004), where the standard deviation is about 5%. The improvement here is possibly due to the use of a 16-bit camera and an energy monitor. A bootstrap resampling approach (Efron 1992; Benedict and Gould 1996) was also employed to estimate the uncertainty in the computed mean values. For every 10th pixel, $10^4$ bootstrap resamples were generated to calculate the relative standard error and relative confidence interval. The mean standard error was approximately $0.18\%$, while a $\pm 0.35\%$ convergence in mean pixel counts was achieved for the $95\%$ confidence interval.

Finally, the resolution of the oxygen point probe used in calibration is $\pm 0.005$ mg/L at 0.4 mg/L and $\pm 0.025$ mg/L at 9 mg/L, as specified by the manufacturer (PreSens). This thus represents the uncertainty on the “ground truth” of the oxygen concentrations that are used for calibration of the LIF.

**Fig. 4:**
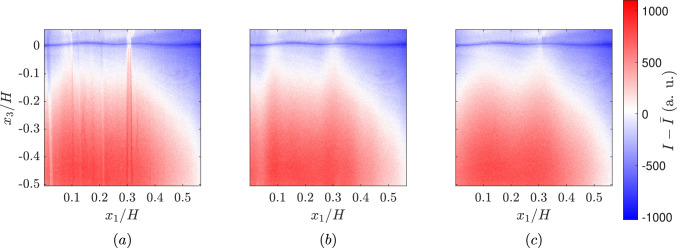
Fluctuating intensity field of** a** a sample raw image,** b** after filtering with FFT method and** c** after filtering with the wavelet-FFT method (15 dB). *I* is the intensity of image in counts, $\bar{I}$ is the mean of the image in counts

To convert pixel intensity counts into physically meaningful concentration units (mg/L) the following steps are followed:

1. **Pulse-to-pulse energy variation:** All LIF images are intensity-corrected to account for pulse-to-pulse energy variation using
  2 $$\begin{aligned} I_{corr}(x_1,x_3) = I_{img}(x_1,x_3)\frac{I_{ref}}{I_{mon}}, \end{aligned}$$
  where $I_{corr}$ is the corrected intensity, $I_{img}$ is the instantaneous image intensity, $I_{ref}$ is the average and $I_{mon}$ is the instantaneous energy counts from the energy monitor.
2. **Reflection removal:** The laser sheet entering from the bottom of the channel is irregularly reflected by the concave and convex surfaces formed by the wavy air–water interface. It appears as bright stripes in the imaging area as seen in  Fig. 4a. To correct this we tried two methods, frequency filtering using the two-dimensional Fast Fourier transform (FFT) method used in Westerweel et al. (2011) and a wavelet-FFT (W-FFT) combined method developed in Münch et al. (2009). Both of these methods are applied only to remove vertical reflections. Attempting to remove other reflections resulted in a significant loss of signal as other reflections are at random orientations and less frequent. In the FFT method, the image is first transformed into the Fourier domain, where the frequencies corresponding to the vertical stripes are masked. The inverse FFT is then applied to transform the image back to the spatial domain, as shown in  Fig. 4b. In the W-FFT method, wavelet decomposition of the image is performed using a 15 dB wavelet (Münch et al. 2009). A 1D FFT is applied to the vertical details of the decomposed wavelet coefficients, which are then multiplied by a Gaussian function to dampen the vertical stripes. The filtered image is reconstructed from the modified wavelet coefficients, as shown in  Fig. 4c.
  The W-FFT method proved to be more effective in identifying and reducing the vertical stripes compared to the FFT method. The final calibration used for further analysis was performed after correcting the reflections using the W-FFT method.
3. **Intensity (counts) to concentration (mg/L):** Calibration is performed by treating each pixel of the camera as a separate sensor (Vanderwel and Tavoularis 2014). The data are filtered in time to reduce noise, but no spatial filtering is applied; applying a spatial filter would not preserve the sharp gradient near the air–water interface. The conversion from intensity to concentration is done using the relation
  3 $$\begin{aligned} C(x_1,x_3) = \left( \frac{C_s-C_b}{I_b(x_1,x_3)-I_s(x_1,x_3)}\right) (I_b(x_1,x_3)-I(x_1,x_3))+C_b, \end{aligned}$$

where $C_s$ and $C_b$ are the saturation and initial concentration from the bulk measurements made using the probe, $I_s(x_1,x_3)$ and $I_b(x_1,x_3)$ are the intensities of the chosen pixel at saturation and the beginning of the experiments. $I(x_1,x_3)$ is the instantaneous fluorescence intensity of the chosen pixel. The values $I_s$ and $I_b$ are calculated as the average of the initial and final 10 images. This results in calibration as shown in Fig.  3b. Note Eq. 3 differs slightly from that used by Herlina and Jirka (2004), as the saturation concentration is taken as the concentration at the end of the experiments, rather than assuming the surface is fully saturated. This is possible here because the full time-series from zero concentration to saturation concentration is acquired.

This calibration method has the advantage that it does not require corrections such as the Beer–Lambert law for laser attenuation since attenuation is accounted for in the pixel-by-pixel calibration. In the current work, we look at DO variation over the range of 0.9 mg/L to 8.5 mg/L. The variation of relative intensity is then plotted against the DO concentration measured by the oxygen probe, as shown in  Fig. 3c. The plot reveals a relative intensity loss of approximately 28% over the range of DO concentrations studied here.

## Results

### Flow conditions in the air and water

The inlet air turbulence was measured using a hot-wire as outlined in Sect. 2.5. The centerline velocity of the air is 6.6 m/s, resulting in a centerline Reynolds number, $Re_H=U_{c} H/\nu = 21700$, where $U_c$ is the mean velocity at the channel centerline. The Taylor microscale, estimated by $\lambda = (\overline{u_1'^2}/(\overline{\partial u_1 /\partial x_1)^2})^{1/2}$, is determined to be 4.3 mm with a corresponding Taylor microscale Reynolds number $Re_\lambda = {u_1}_{rms}'\lambda /\nu =90$. The Kolmogorov microscale $\eta$ is determined to be 0.22 mm. Note the notation herein follows the standard Reynolds decomposition notation, i.e., $u = U + u^{\prime }$ where *u* is the instantaneous velocity, *U* is the mean, and $u'$ is the velocity fluctuations. Furthermore, the standard turbulence double notation is employed where $u'$ is used both for the instantaneous velocity fluctuations and for the standard deviation of the turbulence fluctuations, i.e., $u' = \sqrt{\overline{u'^2}}$, where $\overline{\cdot }$ denotes averaging.

**Fig. 5:**
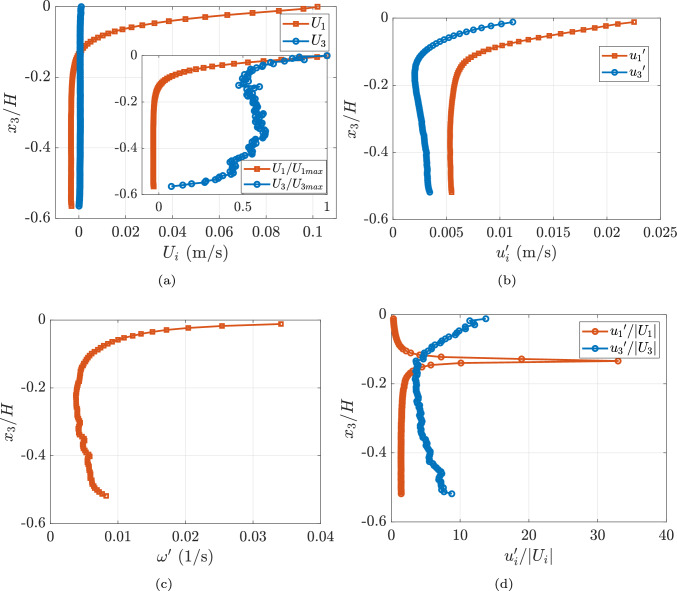
**a** $U_1$ and $U_3$ components of velocity variation with normalized height. The inset in the figure shows normalized $U_1$ and $U_3$ components with respective maximum velocity component variation.** b** Turbulent fluctuation variation with normalized height.** c** Vorticity variation with normalized height.** d** Turbulence intensity variation with normalized height. Profiles are calculated from an ensemble average of over 2577 instances acquired at 0.5 Hz

The turbulent air interacts with the water surface, inducing a velocity field that combines with the velocity field generated by the circulating water pump. PIV measurements were performed in the water, as described in Sect. 2.2. The resulting mean velocity profile is shown in Fig. 5a. In this setup, $x_1$ represents the direction of airflow and is considered positive, while negative $x_3$ corresponds to the direction of gravity (increasing water depth). The average air–water interface is located at $x_3=0$. It is important to note that the bottom boundary layer is not captured, as it lies outside the field of view.

Wind shear also induces wavy structures on the water surface. Although a pump circulates water from one end of the channel to the other at a very low rate, the lower half of the channel is dominated by recirculation. The pump operates at a low flow rate inducing a typical velocity of the order of 0.02 m/s to minimize the time required to achieve a steady velocity profile compared to still water and to reduce PIV particle settling. The water pump induced velocity is an order of magnitude lower than the wind induced water velocity close to the air–water interface and two orders of magnitude lower than the air velocity.

The wind shear leads to higher velocity fluctuations and fluctuating vorticity near the water surface, as seen in Fig. 5b, c. These fluctuations diminish with increasing depth. From Fig. 5a, it is evident that $u_1$ changes sign at approximately $-0.13x_3/H$, indicating the presence of a shear layer. The peak of $u_1^\prime /U_1$ in Fig. 5d indicates the region of the shear layer between the wind-driven flow and the recirculation.

### Simultaneous velocity and concentration field

**Fig. 6:**
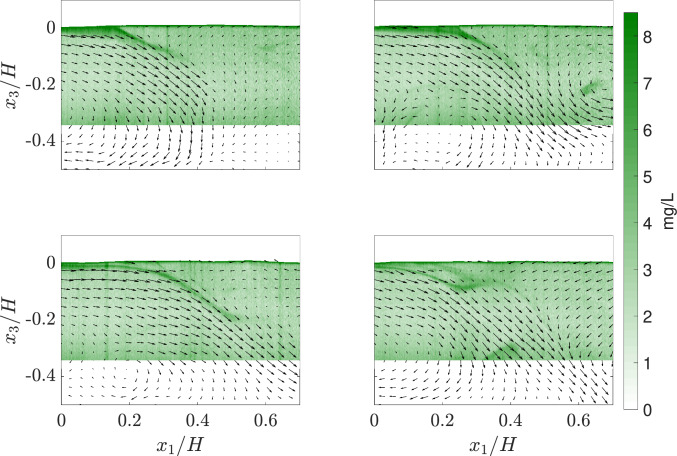
Instantaneous vector fields superimposed on the dissolved oxygen scalar field showing continuous snapshots acquired at 1 Hz. This is taken from a data set with an air velocity of about 4 m/s where the air–water interface is almost flat and the induced velocity has not reached a steady state. The high-intensity (green color) structure indicates higher oxygen concentration than the surroundings (color figure online)

**Fig. 7:**
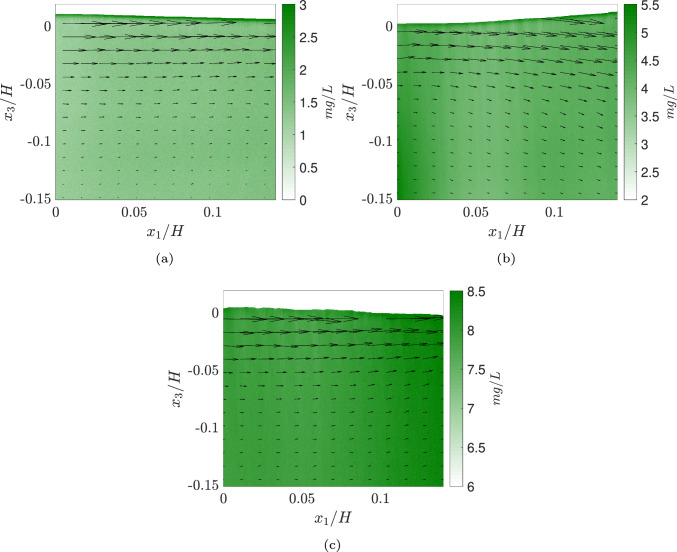
A close view of instantaneous vector fields superimposed on the dissolved oxygen scalar field showing snapshots at** a** t = 1 min,** b** t = 30 min and** c** t = 116 min acquired at 0.5 Hz. This is taken from a data set with an air velocity of about 6.6 m/s where the air–water interface is wavy and the induced velocity has reached a steady state. The high-intensity (green color) indicates higher oxygen concentration (color figure online)

The instantaneous oxygen LIF images, superimposed with velocity vectors, are shown in Figs. 6 and 7. The oxygen LIF images are calibrated into meaningful concentration units (mg/L). Dissolved oxygen from the atmosphere creates a high concentration layer at the water surface. In the instances shown in Fig. 6, where the water surface is almost flat and the flow has not reached a steady state, it can be seen that the vertical flow carries the high DO concentration layer into the flow, appearing as structures. These structures shear off from the surface and mix with the bulk of the water. At higher air velocities and steady-state flow, we observe a high DO concentration interface but structures are not seen to directly mix with the bulk of water (Fig. 7). In these cases, the $u_1$ component is strong near the interface, and $u_3^\prime$ becomes the driving factor in mixing from the surface into the bulk. This is further discussed in Sect. 4.5. Furthermore, as shown in Fig. 7a–c, the difference in concentration between the surface and the bulk reduces as the bulk concentration approaches saturation.

### Spatiotemporal concentration variation

**Fig. 8:**
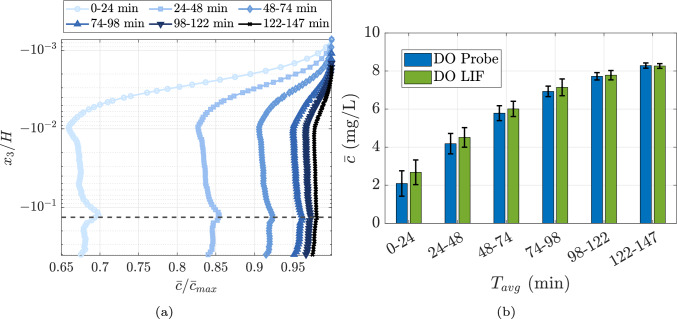
**a** Normalized DO concentration variation with normalized height with each line indicating a 24 min average in time,** b** A comparative plot between DO probe measurements and oxygen LIF measurements

The experiment spans approximately 147 min, with the DO concentration increasing from an initial value of 0.9 mg/L to about 8.5 mg/L. The flow conditions of the air and water are discussed in Sect. 4.1. Figure 8a illustrates the variation in normalized dissolved oxygen (DO) concentration as a function of normalized height. Each line represents the average concentration profile over a 24.5-minute interval (corresponding to 735 images), normalized by the maximum concentration, which corresponds to the surface concentration.

Initially, the surface DO concentration is approximately 35% higher than the bulk region concentration. As time progresses, the overall DO concentration in the system increases, with the bulk DO concentration gradually approaching the surface concentration, as seen in the 122–147 min interval. Near the surface, the DO concentration decreases sharply within the first 0.1*H* of depth. The DO concentration remains nearly constant before increasing slightly, forming a local maxima near $-0.13H$, marked with a broken black line. This maxima corresponds to the turbulence intensity peak observed in Fig. 5c, highlighting the influence of flow features such as shear layers on the scalar mixing dynamics. As the system approaches saturation, this peak diminishes. Below the shear layer, the DO concentration becomes comparable to that of the region above it, with a slight decrease observed at greater depths. An important take-away from these observations is that the profile is not constant in time, and thus, when the measurements are performed (or at what state of saturation the bulk fluid is in) matters.

Figure 8b compares the average DO concentrations obtained from LIF measurements and bulk measurements from the probe. The LIF measurements capture data from the top 40% of the channel height, while the probe measurements represent a single point at 50% of the channel depth. Given this difference, a close match between the two methods is not expected during the initial stages of the experiment. However, as the system approaches saturation, the probe measurements align closely with the LIF data. The standard deviations shown in the plots represent variations within each 24-minute average and should not be interpreted as measurement errors.

### Concentration fluctuations

**Fig. 9:**
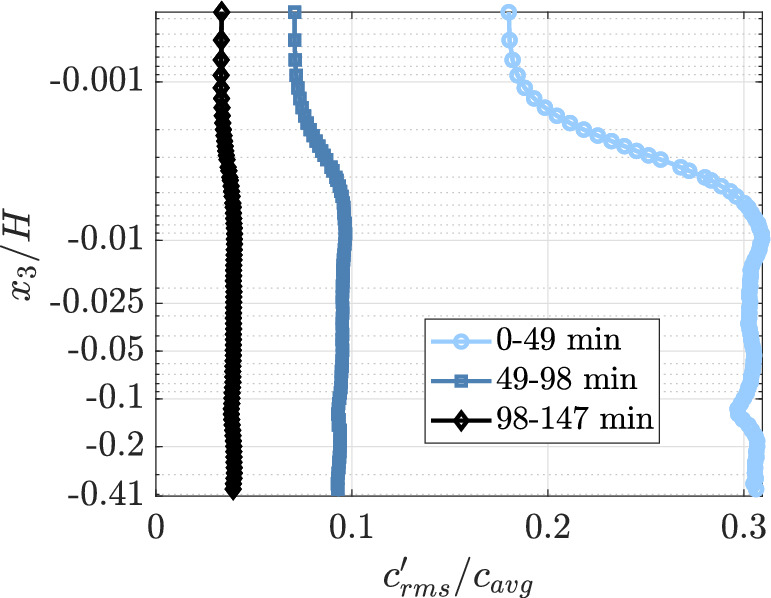
Normalized concentration fluctuation plotted against normalized height in a semi-log $x_3$ axis with three averages from least to saturation concentration

The concentration fluctuations are calculated for three time intervals, each about 49 min using equation $c^\prime = \sqrt{\overline{(c-\bar{c})^2}}$, where $\bar{c}$ is the average for each 49 min interval considered. The variation of fluctuations in height is shown in Fig. 9 in normalized units. The $\bar{c}(x_3)$ used to normalize the fluctuations is the spatial mean of $\bar{c}$ that still varies with height.

The concentration fluctuations near the interface are smaller than the bulk fluctuations as the DO concentration variation at the interface is smaller. This trend is similar to that reported in Herlina and Jirka (2004), although they observed zero fluctuation at the interface. The difference arises because we do not assume the surface to be saturated in all instances. As the system approaches saturation, the total magnitude of normalized fluctuation decreases. Additionally, toward saturation, the fluctuations become nearly uniform throughout the height of the water. This again points to the importance of when the measurements are performed during the re-aeration process and thus the importance of the bulk concentration of DO as it directly influences the mechanics of the problem.

### Turbulent scalar transport

With simultaneous velocity and concentration measurements in water, turbulent scalar transport can be calculated. Since the velocity and DO concentration data have different resolutions, a smoothing filter is applied, and the concentration field is down-sampled to match the velocity data resolution. The scalar flux is then calculated as $\langle u_i^\prime c^\prime \rangle ~=~\sqrt{\overline{(u_i^\prime c^\prime )^2}}$, where $u_i^\prime =u_1^\prime$ for horizontal flux and $u_i^\prime =u_3^\prime$ for vertical flux.

**Fig. 10:**
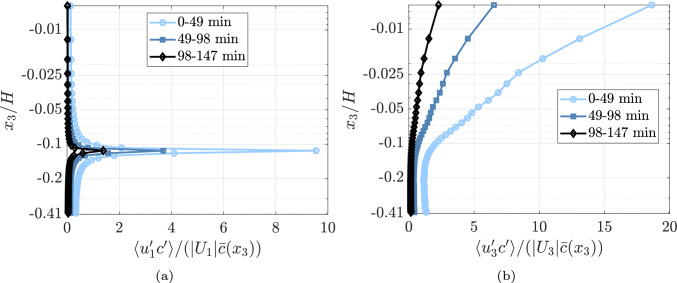
Normalized scalar flux plotted against normalized height with a mean calculated for each 49 min interval. Here also mean is calculated for 49 min intervals

Figures 10a, b presents the normalized horizontal and vertical fluxes, where normalization is performed using the average concentration and respective absolute mean velocity. These plots illustrate the influence of velocity fluctuations relative to the mean flow in scalar transport. As shown in Fig. 10b, the flux carried by vertical velocity fluctuations near the interface is substantial, being an order of magnitude higher than the horizontal flux. The peak observed in Fig. 10(a) is due to normalization by the absolute mean $U_1$ velocity and corresponds to the shear layer discussed earlier. This might suggest that horizontal flux due to $u_1^\prime$ is significantly higher in the shear region due to a very small $U_1$ component. Additionally, as the water approaches saturation, the magnitude of $c^\prime$ decreases, leading to a corresponding reduction in both horizontal and vertical fluxes.

## Challenges and considerations

From an experimental perspective, there are a few challenges, pitfalls, and considerations that should be taken into account when performing similar measurements. For the sake of the reader, we list some here:

- It was observed during the experiments that PBA can photobleach when excited with high power and frequency. This is particularly seen in low-velocity flow when there is the potential for the same water molecules to be excited multiple times. The resulting photobleached dye creates structures resembling high DO structures because the photobleached dye is still convected by fluidic motions.
  This phenomenon was not explicitly reported in the literature previously, perhaps because the issue was exasperated in the present study by newer, more powerful lasers. Nonetheless, it should be kept in mind that just because structures are seen, it does not mean they represent DO.
- Test experiments were done with tap water. However, using deionized (DI) water, the signal-to-noise ratio of the oxygen LIF was significantly improved. The turbidity in the water can greatly reduce the signal. This may cause issues when up-scaling the approach.
- When the flow is advecting and the free surface is allowed to deform, the laser cannot be fired through the interface, i.e., from the air to the water. There is significant scattering and the generation of high- and low-intensity streaks that are purely optical, and effectively a random result of the instantaneous surface, and very difficult to remove. Even when the laser is fired from beneath the interface, internal reflections cause problems as demonstrated in Fig. 4.
- The laser light at the UV wavelength attenuates rapidly in the water (both for DI and tap water, but the situation is more severe for tap water). In the present facility, the laser passes through approximately 50 mm of water to reach the measurement area. We presently believe that this cannot be significantly changed (dimensionally) with equipment such as that employed herein (which is relatively high energy per pulse at the UV wavelength).
- The time measurements are performed while progressing from zero DO concentration to saturation concentration (or alternatively, the value of the bulk DO concentration) which has an effect on the measured scalar fluxes, and averaging over a long period is in effect averaging a transient phenomenon.

## Conclusions

This study provides a comprehensive investigation into oxygen transfer across a deforming air–water interface, leveraging the simultaneous measurement capabilities of particle image velocimetry (PIV) and laser-induced fluorescence (LIF). The experimental setup successfully captured the dynamic interplay between airflow, interface deformation, and dissolved oxygen (DO) transport. The calibration of the LIF intensity field benchmarked against optical oxygen probe measurements, ensured the accuracy and reliability of the DO concentration data.

The simultaneous capture of DO and velocity fields provided critical insights into the coupling between turbulent flow structures and oxygen distribution. The spatial and temporal variations in DO concentration were effectively visualized, illustrating the influence of turbulent mixing on oxygen dissolution. The spatial evolution of the DO concentration revealed a steep gradient near the interface that diminished with depth. The temporal evolution showed that the concentration difference between the surface and bulk regions was approximately 35% during the initial phase of the experiment, reducing to less than 5% as the system approached oxygen saturation. Concentration fluctuations were lower near the interface than in the bulk, with a consistent decline in fluctuations as saturation was achieved. The vertical and horizontal flux normalized by the average concentration and respective absolute mean velocity indicates that the vertical fluctuations are the driving factor for the transport of the scalar into the water compared to the mean vertical velocity. On the other hand, though horizontal fluctuations are of higher magnitude, the mean flow is the driving factor in the horizontal direction. A significant result here is that the fluxes change significantly in time relative to the total saturation state of the system, and thus,* when* in this process measurements are performed should be rigorously documented. This was not always the case in previous investigations, but few endeavored to measure across the full re-aeration process. This should perhaps be a focus of future studies and understanding how the mechanics change based on the saturation level may inform why there is such diversity in gas transfer models—perhaps the driving mechanisms change with the bulk concentration, and this is why one model does not appear to be able to predict all results in the literature.

## Data Availability

All vector and concentration fields from the present investigation as well as all other data supporting the plots presented herein are publicly accessible at doi: 10.18710/RECIXN
